# Urinary Tract Infection Caused by Lactococcus garvieae in a 75-Year-Old Male Patient With a Complex Medical History

**DOI:** 10.7759/cureus.80608

**Published:** 2025-03-15

**Authors:** Baker Edrees, Muhammad Ali Khalid, Munir Shah, Rida Ghani

**Affiliations:** 1 Internal Medicine, American University of Antigua College of Medicine, Coolidge, ATG; 2 Internal Medicine, Western Reserve Health Education, Warren, USA

**Keywords:** complicated uti, lactococcus garvieae, lower urinary tract infection, recurrent uti, urinary tract infection treatment

## Abstract

*Lactococcus garvieae*, a Gram-positive bacterium traditionally associated with infections in fish, has been increasingly recognized as a rare pathogen in human infections, including urinary tract infections (UTIs). We present a case of a 75-year-old male patient with pyohydronephrosis and a complex medical history who was diagnosed with a UTI caused by *L. garvieae*. The patient was successfully treated with a combination of intravenous ampicillin/sulbactam followed by oral linezolid, resulting in the resolution of symptoms. This report also compares this case with other documented cases of *L. garvieae* infections, highlighting its pathogenic mechanisms, clinical manifestations, treatment strategies, and the need for heightened awareness of this pathogen.

## Introduction

*Lactococcus garvieae* is a Gram-positive coccus primarily known as a pathogen in fish, particularly in farmed aquatic organisms (aquaculture). However, in recent years, *L. garvieae *has been increasingly implicated in human infections, including endocarditis, biliary tract infections, and urinary tract infections (UTIs), and often in individuals with underlying health conditions or immunocompromised states [1-9]. UTIs caused by *L. garvieae* are particularly rare, with only a few cases documented in the literature. This report presents a case of a UTI caused by *L. garvieae* in an elderly male patient with a complex medical history, underscoring the importance of recognizing this pathogen in atypical presentations. 

Diagnosis of an infection with *L. garvieae* involves microbiological culture and identification. Blood cultures are essential for detecting bacteremia or endocarditis. The bacterium can be identified using biochemical tests, such as catalase and oxidase tests, and confirmed by molecular methods like 16S rRNA gene sequencing or Matrix-Assisted Laser Desorption Ionization - Time of Flight (MALDI-TOF) mass spectrometry [4]. Depending on the susceptibility profiles, treatment typically involves antibiotics such as the penicillin family, vancomycin or gentamicin [4-9]. For instance, *L. garvieae* isolates from bovine mastitis were found to be susceptible to penicillin, ampicillin, amoxicillin-clavulanic acid, imipenem, ceftiofur, enrofloxacin, and marbofloxacin [7]. All isolates had virulence genes coding for collagenase, fibronectin-binding protein, glyceraldehyde-3-phosphate dehydrogenase, superoxide dismutase, and nicotinamide adenine dinucleotide + hydrogen (NADH) oxidase. Most isolates had lsaD and mdtA antimicrobial resistance genes [4-7].

*L. garvieae* is thus a versatile pathogen with a broad host range. It causes various diseases in aquatic, bovine, and human hosts. Its diagnosis and treatment require specific microbiological and antibiotic susceptibility testing. This case highlights the differential diagnosis of *L. garvieae* in patients with suspected complex UTI and underscores the need to consider screening for this pathogen when primary differentials are ruled out. Given its genomic features, including virulence and resistance genes, *L. garvieae* may contribute significantly to pathogenicity.

## Case presentation

**Figure 1:**
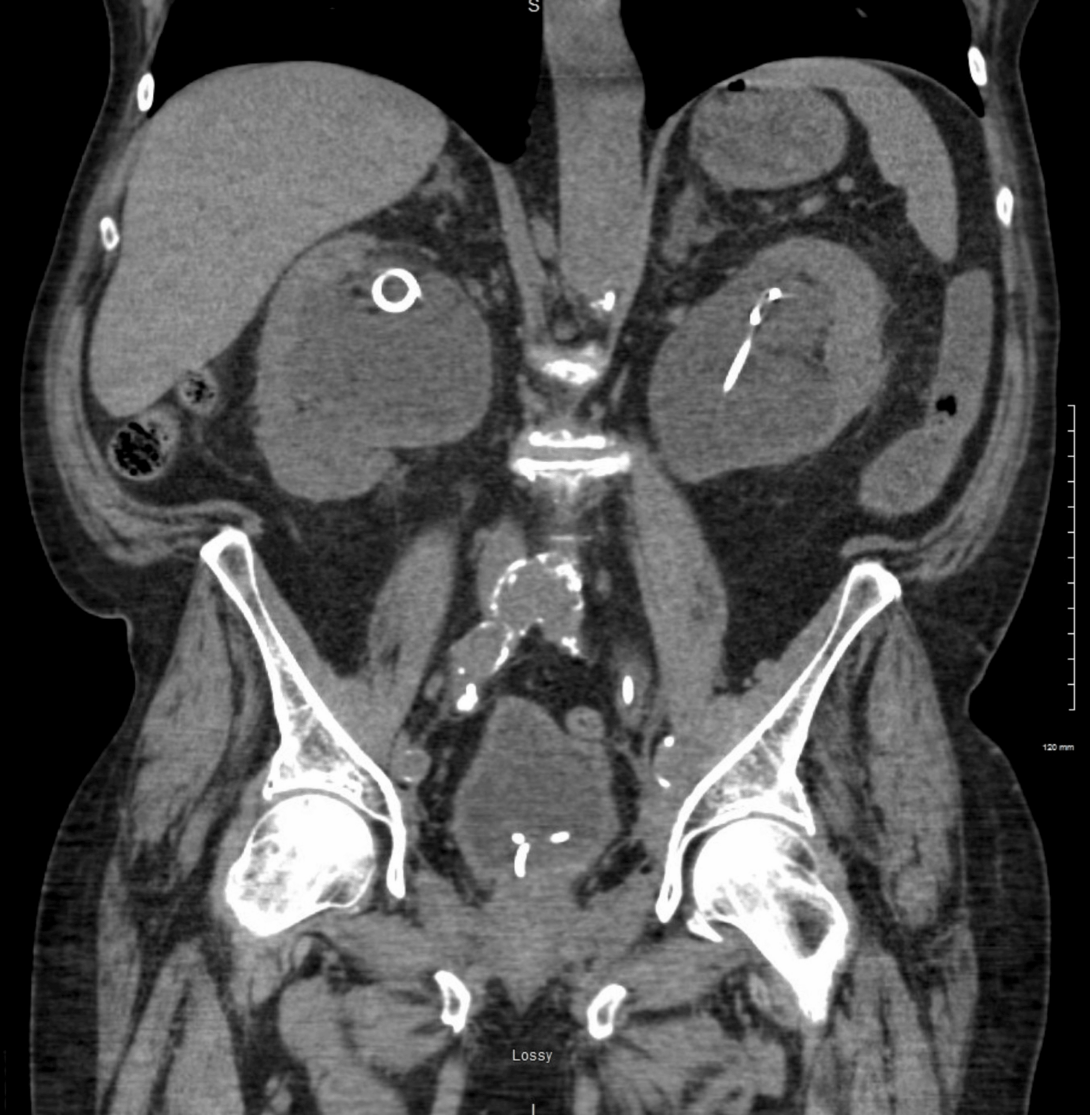
CT scan showing severe bilateral hydroureteronephrosis and the presence of ureter stents

A 75-year-old male patient with a past medical history of chronic kidney disease (CKD), chronic obstructive pulmonary disease (COPD), sick sinus syndrome, urethral strictures secondary to prostate cancer radiation therapy (requiring periodic J-stent replacement), and a history of rectal adenocarcinoma (current status post-resection) presented to the emergency department. He complained of 10/10 constant, sharp pain localized to the hypogastric region, which was associated with vomiting and fever. The patient denied any urinary symptoms. However, his symptoms had persisted for several days, prompting medical evaluation. A CT scan of the abdomen and the pelvis revealed bilateral hydroureteronephrosis [Figure 1]. A Foley catheter was placed, and the patient received a single dose of ceftriaxone in the emergency department.

The patient was admitted. He was afebrile and hemodynamically stable. Urinalysis showed the presence of red blood cells (RBCs) and white blood cells (WBCs), and was positive for esterase [Table 1].

**Table 1:** Urinalysis HPF = High power field, E. U. = Ehrlich units, LPF = Low power field, Tntc = Too numerous to count, H = High values, A = Abnormal values

Urine content	Results	Normal (range if applicable)
Urine color	Straw color	Yellow
Urine clarity	Turbid	Clear
Urine pH	6	5.0-8.0
Urine specific gravity	1.025	1.005-1.030
Urine protein (mg/dL/24 hrs)	100 (H)	None seen
Urine glucose (mg/dL)	None seen	None seen
Urine ketones (mg/dL)	None seen	None seen
Urine blood (mg/dL)	Large (A)	None seen
Urine nitrate (mg/dL)	None seen	None seen
Urine urobilinogen (E.U./dL)	0.2 (A)	None seen
Urine bilirubin (mg/dL)	None seen	None seen
Urine leukocyte esterase (mg/dL)	Large (A)	None seen
Urine RBCs per HPF	6-10 (A)	0-2
Urine WBCs per HPF	Tntc (A)	0-5
Urine squamous epithelial cells per HPF	None seen	0-5
Urine transition epithelial cells per HPF	0-5	None seen
Calcium oxalate crystals per HPF	Few (A)	None seen
Amorphous crystals per HPF	Few (A)	None seen
Urine bacteria per HPF	Many (A)	None seen
Hyaline casts per LPF	None seen	None seen
Urine mucus per LPF	Present (A)	None seen

The urine culture grew bacteria consistent with *L. garvieae* [Table 2]. 

**Table 2:** Urine cultures

Sample location	Final culture result
Urine, clean catch	*Lactococcus garvieae, *no Gram negative rod
Urine, Kidney tap right	Alpha hemolytic strep, not Gram positive type D
Urine, Kidney tap left	Alpha hemolytic strep, not Gram positive type D

The patient was diagnosed with pyohydronephrosis, a suppurative kidney infection secondary to obstruction, and was started on a four-week course of intravenous ampicillin-sulbactam (Unasyn), followed by four weeks of oral linezolid (Zyvox) to specifically target *L. garvieae. *Throughout the admission, the patient remained afebrile and hemodynamically stable. A urinary voiding trial was successful, allowing for the removal of the Foley catheter to prevent further complications. The patient responded well to treatment, demonstrating significant clinical improvement. There was symptom resolution by day four and progressive resolution of hydroureteronephrosis.

## Discussion

This case highlights several key aspects of *L. garvieae *as an emerging pathogen in human infections. Its pathogenesis in UTIs involves its ability to adhere to uroepithelial cells, form a capsule, and produce extracellular enzymes such as proteases and hemolysins [10]. These virulent factors contribute to the bacterium’s ability to cause tissue damage and evade the host immune response [10]. In this patient, urethral strictures and periodic stent replacements likely created an environment conducive to bacterial colonization and infection. The ability of *L. garvieae* to form a capsule may have played a significant role in the persistence of the infection, particularly in the presence of indwelling devices such as J stents. While such UTIs are rare, a literature review reveals a few documented cases that provide valuable insights into this pathogen's clinical presentation and management.

A case by Amarasinghe et al. [4] reported *L. garvieae *bacteremia associated with adenocarcinoma, illustrating its opportunistic nature in immunocompromised patients. Bacteremia developed following a suspected breach of mucosal barriers [4], emphasizing the need for vigilance in managing these infections in patients with cancer. This is particularly relevant to our case, given the patient’s history of rectal adenocarcinoma and ureteral stent placement, which may have contributed to the mucosal barrier disruption.

Colussi et al. documented the first human UTI caused by *Lactococcus petauri*, another species within the *Lactococcus* genus [5]. This report expands the clinical spectrum of *Lactococcus* species as human pathogens. It suggests vigilance in identifying these unusual pathogens, especially in patients with recurrent or refractory UTIs [5]. In contrast, Malek et al. described a case of infective endocarditis caused by *L. garvieae*, highlighting the bacterium’s potential to cause severe systemic infections beyond the urinary tract [7]. The case involved a patient with a history of mitral prosthetic valve, suggesting that *L. garvieae* may preferentially infect individuals with preexisting cardiac conditions. This case emphasizes the importance of considering it as a potential cause of infection in patients with complex medical histories, like our patient who had multiple comorbidities [7].

Tariq et al. reported a similar case involving a 74-year-old female patient with recurrent UTIs who presented with fever and dysuria [8]. Urine cultures confirmed *L. garvieae*, and the patient was successfully treated with ampicillin. In our case, the patient had underlying urinary tract abnormalities, which likely contributed to the infection [8]. Additionally, Woolery reported an acute lower UTI caused by *L. garvieae* in a patient without significant predisposing factors [9]. This case is notable as it suggests that *L. garvieae* can cause UTIs even in individuals without the complex medical histories observed in other cases. In this case, the successful treatment with ceftriaxone further underscores the need for antibiotic susceptibility testing in guiding treatment [9].

These comparative cases emphasize the importance of recognizing *L. garvieae *as an emerging human pathogen, particularly in patients with complex medical histories, immunosuppression, or urinary tract abnormalities. The diversity in clinical manifestations and outcomes observed in these cases highlight the need for early recognition, appropriate culture, sensitivity testing, and targeted antibiotic therapy in managing infections by the *Lactococcus* genus. 

## Conclusions

This case of a UTI caused by *L. garvieae* in a 75-year-old male patient with a complex medical history adds to the growing evidence of it being an emerging human pathogen. Given its rarity in human infections, clinicians should maintain a high index of suspicion in UTIs with atypical presentations or multiple predisposing factors. The successful outcome in this case underscores the importance of timely diagnosis and targeted antibiotic therapy. *L. garvieae* should be included in the differential diagnosis of UTIs, particularly in patients with complex medical histories and indwelling devices. Its ability to form biofilms may contribute to the persistent infections, necessitating prolonged or targeted antibiotic regimens. Early culture and sensitivity testing are crucial for guiding effective treatment. Future research should further elucidate the pathogenic role of *Lactococcus* species and establish management guidelines for these rare infections.
